# Economic elasticities of input substitution using data envelopment analysis

**DOI:** 10.1371/journal.pone.0220478

**Published:** 2019-08-08

**Authors:** Noah J. Miller, Jason S. Bergtold, Allen M. Featherstone

**Affiliations:** Department of Agricultural Economics, Kansas State University, Manhattan, Kansas, United States of America; Tohoku University, JAPAN

## Abstract

The use of elasticities of substitution between inputs is a standard method for addressing the effect of a change in the mix of inputs used for production from a technical or cost standpoint. Most estimation methods use parametric production or cost functions or frontiers to estimate these elasticities. A potentially useful nonparametric alternative is data envelopment analysis (DEA). The purpose of this paper is to derive elasticities of input substitution for both technical and cost frontiers using DEA, extending the use of this approach in the field of economics and associated fields. The paper provides derivations for both Hicksian (production and cost frontier) and Morishima (cost frontier) elasticities of input substitution, as well as a parsimonious method for estimating them using DEA. The derivations are presented using an agricultural example form Kansas, USA.

## Introduction

Agricultural production is characterized by increases in productivity and frequent changes in price. For a firm wanting to maximize profit (or minimize cost), an understanding of the tradeoff, or substitutability, of one input for another as prices change is essential. The derivation of elasticities of substitution is the standard method for addressing the effect of a change in the inputs used for production from a technological or cost perspective [1]. From the technological perspective (using a production function), this measurement shows how a per unit change in the marginal rate of technical substitution alters the ratio of inputs, while maintaining a fixed level of output. In the case of a cost function, the elasticity of substitution shows how a shift in input prices shifts the ratio of inputs. More generally, it relates a percentage change in the ratio of inputs being used to an incremental increase in the ratio of the marginal products of the inputs (the ratio of input prices) [1].

Since [2] derived the elasticity of substitution to describe the effect that a change in the capital to labor ratio has on income distribution, several attempts have been made to generalize the Hicksian result to the case of more than two inputs [3–7]. Of these, the Morishima elasticity of substitution (MES) has become the conventional method [8–9]. Of the alternative measurements used in the literature, the Allen-Uzawa elasticity of substitution has been criticized for not being a direct measurement of the curvature of the isoquant, while the McFadden elasticity has been criticized for not allowing optimal adjustment of inputs in response to changes in input prices [10]. The MES is a direct measure of the curvature of an isoquant that flexibly captures the effect on input shares from a change in input price or input quantity.

Statistical estimation of any of these elasticities of substitution from data is usually accomplished by estimating a parametric production or cost function. For a study with only cross-sectional data, estimating an elasticity of substitution may prove problematic for the cost function, due to limited relative price variability [11–13]. In such a situation, a nonparametric alternative, such as Data Envelopment Analysis (DEA), may be a useful approach. Estimating elasticities using DEA offers some advantages. DEA methods require minimal assumptions about the underlying production technology of the firms being observed and allows for individual estimates of elasticities of substitution for each firm in the sample. [14] and [15] emphasize that DEA can give better information on trade-offs (between inputs and outputs) than regression analyses. [16] and [17] showed that DEA provides a robust estimator of individual level economic measures at the frontier (technical or cost) and that DEA’s empirical performance in approximating underlying technology is less effected by distributional assumptions than parametric approaches. [18] showed how DEA can derive marginal rates of substitution for both efficient and inefficient firms or decision making units, which are needed for estimation of elasticities of substitution.

[14] examined the estimation of marginal rates from DEA models, including the marginal rate of substitution between two inputs, along DEA frontiers using directional derivatives. These methods were not extended to the estimation of elasticities of substitution. [19] provided a method for estimating elasticities of substitution for a slacks-based technical efficiency problem using DEA under variable returns to scale (VRS). However, this study presents a general elasticity of substitution that does not directly take into account changes in the ratio of marginal products or prices between the two inputs being examined. The authors did not derive the Hicksian elasticity commonly reported in the economic literature and did not extend their estimation to the cost efficiency problem.

More recently, [20] provided a general framework for calculating directional derivatives useful for estimating elasticities of scale and marginal rates within a DEA framework (further explored by [21]). [22] examine methods for estimating returns to scale measures using directional derivatives with DEA. In addition, [20] provided a general framework for estimating directional elasticity measures (e.g. scale elasticity) and marginal rates (e.g. marginal rate of substitution) at the extreme points of the DEA frontier. This work has been extended to be able to include undesirable output [23]. [24] examined methods to develop efficiency approaches to capture constant elasticity of substitution and transformation technologies. These researchers did not explicitly examine or report elasticities of substitution commonly used in the production economics literature. [1] emphasizes that marginal rates, including marginal rates of substitution, may be of limited practical use. Marginal rates of substitution only examine the physical substitutability between two inputs holding all other inputs constant. In general, economists are also interested in how all inputs adjust or respond when the level of a particular input changes. This observation extends to simple elasticities of substitution based only on the marginal rate of substitution. Past DEA literature has primarily focused on estimation of marginal rates and elasticities for technical efficiency, while the majority of economic literature focuses on elasticities of substitution that are based on the use of cost functions [9]. There exists a need to examine elasticities of substitution for cost efficiency DEA problems.

The purpose of this article is to provide a method for estimating Hicksian and Morishima elasticities of substitution for the technical production and cost efficiency DEA models under VRS. These elasticity measurements have yet to be encountered in the DEA literature. In addition, estimation of Morishima elasticities of substitution for the cost efficiency DEA problem allows all inputs to change in response to a change in a given input, allowing for additional useful estimates of elasticities of substitution. In this way, the current study extends the work of [16], [18], [19], [20] and others. Unlike more recent efforts to calculate (directional) marginal rates and elasticities using DEA along the entire efficient frontier, a limitation of the methods presented here is the focus on estimation of elasticities of input substitution on the relative interior of the facets of the efficient frontier, using the optimal dual variables [25]. We leave the estimation of input elasticities of substitution presented here at the extreme points of the frontier (for efficient firms) as future research. This work provides a first attempt at estimating these specific elasticities using DEA.

## Data envelopment analysis

This study builds upon the DEA methods examining technical and cost (economic) efficiency as presented by [26–31]. This section of the paper presents the technical and cost efficiency DEA problems used to derive elasticities of substitution. All the DEA models examined are estimated using an input-oriented approach, assuming variable returns to scale (VRS) for generalizability.

### Technical efficiency problem

The following input-oriented technical efficiency model is used to evaluate the relative efficiency of a group of firms [28–29]:

$${min}_{\theta_{0}}\{\theta_{o}:\;\lambda^{\prime }x_{k}\leq \theta_{o}x_{k,o}\;\forall \;\text{k}\;(\to v_{k});\lambda^{\prime }y_{m}\geq y_{m,o}\forall \;m\;(\to u_{m});{\;e}^{\prime }\lambda =1\;({\to u}_{0});\lambda \geq 0\},$$ (1)

where ***x***_***k***_ denotes a (*n* x 1) vector of the *k*^th^ input, ***y***_***m***_ denotes a (*n* x 1) vector of the *m*^th^ output, ***e*** denotes a vector of ones, ***λ*** denotes a vector comprised of weights associated with each firm in the sample, *k* = 1,…,*K* denotes the index for inputs, *m* = 1,…,*M* denotes the index for outputs, and *n* = 1,…,*N* denotes the index for firms or decision-making units (DMUs). The terms in parentheses after each set of constraints are the associated dual variables for the given set of constraints. When there is only one output being considered the efficient production frontier can be viewed as a production function [32].

The objective of DEA in problem (1) is to estimate technical efficiency, *θ*_*o*_ ∈ [0, 1], of each firm relative to all other firms in the sample, by choosing the vector of weights, ***λ***. Technical efficiency, *θ*_*o*_, is a radial measurement, describing the distance between any individual firm and the efficient frontier, with *θ*_*o*_ equal to 1 characterizing a firm that resides on the efficient frontier. The first and second sets of equations constrain a firm’s composite (efficient) input level to be less than or equal to the technically efficient input level and a firm’s composite (efficient) output level to be greater than or equal to the firm’s given level of output. The input and output weight vectors, ***v***_***k***_ and ***u***_***m***_ represent shadow prices (multipliers or dual variables) to the first and second constraints. The input and output weights identify the relative importance of inputs and outputs in determining a firm’s technical efficiency. The third constraint is a convexity condition allowing for variable returns to scale [33]. The elasticities of substitution are derived from this technical efficiency model.

The vectors of shadow prices ***v***_***k***_ and ***u***_***m***_ arise from the dual of the input-oriented technical efficiency model given by problem (1):

$${max}_{\theta_{D,0}}\{\theta_{D,0}=u^{\prime }y_{o}-u_{0}:v^{\prime }x_{o}=1;u^{\prime }y_{n}-v^{\prime }x_{n}-u_{0}\leq 0\;\forall \;n\text{;}\;v,\;u\geq 0\}.$$ (2)

The term *u*_*0*_ is unrestricted in sign and can be interpreted as a measure of scale efficiency along a particular facet of the frontier [14].

### Cost efficiency problem

Alternatively, in the situation where input prices and costs are available, DEA can be applied to assess cost or overall economic efficiency. Cost efficient firms are defined as those that are technically efficient and also exhibit allocative efficiency [33]. Allocative efficiency is the degree to which a firm or decision-making unit minimizes cost along the technically efficient frontier. The cost efficiency DEA problem is given by:

$${min}_{\theta_{C}}\{\theta_{C}=w^{\prime }z:z_{k}-\lambda^{\prime }x_{k}\geq 0\;\forall \;k\;(\to v_{k});\lambda^{\prime }y_{m}-y_{m,o}\geq 0\;\forall \;m\;(\to u_{m});\;e^{\prime }\lambda =1\;({\;\to u}_{0}\;);\lambda ,z\geq 0\},$$ (3)

where ***w*** is a (*k* x 1) vector of input prices and ***z*** is a (*k* x 1) vector of cost minimizing inputs. The objective function chooses ***z*** and ***λ*** that reduce the firm’s costs, where ***y***_***m***_ denotes the *m*^th^ output, and ***λ*** denotes the peer-group weights. Cost efficiency is calculated as $\frac{w^{\prime }z}{w^{\prime }x_{o}}$ or the distance between the cost of the firm’s actual choice of inputs, ***x***_***o***_, and the cost minimizing level of inputs, ***z*** [33]. Again, the input and output weight vectors, ***v***_***k***_ and ***u***_***m***_ are shadow prices (multipliers or dual variables) to the first and second constraints. Here, they identify the relative importance of inputs and outputs in determining a firm’s cost efficiency (i.e., ***v***_***k***_ represents the marginal cost of an increase in input use, while ***u***_***m***_ represents the marginal cost of an increase in output production). Following [14], *u*_0_ can be interpreted as a measure of scale efficiency. The first constraint requires that the *k*^th^ cost minimizing input to be less than or equal to the composite *k*^th^ input or level of input on the cost frontier in closest proximity to the firm. The second constraint requires the composite output to be greater than or equal to the *m*^th^ output of the firm of interest. The third constraint represents the convexity condition that allows for variable returns to scale.

The dual problem to the cost efficiency problem presented above, provides the linkage to the shadow prices (dual variables) for the primal cost efficiency problem. This problem is given by:

$${max}_{\theta_{DC}}\{\theta_{DC}=y_{0}^{\prime }u-u_{0}:\;v\leq w\;\forall \;k;u^{\prime }y_{n}-v^{\prime }x_{n}-u_{o}\leq 0\;\forall \;n;\;v,\;u\;\geq 0\}$$ (4)

The shadow prices for the technical efficiency formulation in problems (1) and (2) are not necessarily equivalent to the shadow prices for the cost efficiency formulation in problems (3) and (4).

## Economic elasticities of substitution

This section examines the derivation of Hicksian and Morishima elasticities of substitution. The estimation of these elasticities for a cost minimizing firm occurs on the firm’s respective cost and technical frontiers. A drawback of using DEA is that the cost and technical frontiers are piecewise linear in nature. The convex hull that represents the frontier in each case has “kinks”, or vertex points, where the frontier is of measure zero (e.g. a finite set of isolated points of discontinuity) and nondifferentiable [34]. It is at these kinks that economic measures of interest are often calculated, but nondifferentiability at these points results in a lack of unique multipliers or shadow prices (and in turn, associated economic measures that use these multipliers) [34]. While solutions to these problems have been proposed using directional derivatives, the resulting estimates may still be non-unique [14,20] or difficult to estimate in larger dimensional problems. Estimates at these kinks will invariably use the directional derivatives along the intersecting segments. [34] provide a methodology for calculating directional derivatives at the vertices of a DEA frontier, and [35] and [20] show how directional derivatives can be used to calculate marginal rates and elasticity measurements for a technology exhibiting VRS features.

Given the primary interest in the estimates of economic measures (e.g. elasticities of substitution) along each of the facets of the convex hull representing the frontier, an alternative approach may be to estimate the corresponding economic measures for each of the inefficient firms at their projections onto the frontier that exist along the interior of a relative facet of the frontier. That is, to estimate economic measures of interest for the composite firm on the frontier, based on the resulting composite inputs, ${\hat{x}}_{k}=\lambda^{\prime }x_{k}$, and outputs, **${\hat{y}}_{m}=\lambda^{\prime }y_{m}$**. Economists are usually interested in optimal behavior of firms, which suggests focusing on behavior on the frontier.

A particular property of the DEA models is the marginal rate of substitution (MRS) between two inputs is the same at the point of inefficiency as on the projected frontier. [33] show that the projection for an inefficient firm on the frontier using the composite inputs ${\hat{x}}_{k}=\lambda^{\prime }x_{k}$ and ${\hat{y}}_{m}=\lambda^{\prime }y_{m}$ is technically efficient (i.e. $\hat{\theta }=1$, where $\hat{\theta }$ is the technical efficiency problem for the inefficient firm for the projection onto the frontier). By duality, then there exists a set of associated optimal multipliers $\hat{v\;},\;\hat{u}$, and ${\hat{u}}_{0}$ that solve the associated dual multiplier problem (2) [33]. Let ***v*, *u***, and *u*_0_ represent the optimal multipliers for the inefficient firm in problem (2) and let the level of efficiency for the firm be given by *θ*. By complementary slackness, ***v***(***Xλ*** − *θ****x***_**0**_) = **0**, which implies that ***vXλ*** = ***v****θ****x***_**0**_. Rearranging terms gives $\frac{v}{\theta }X\lambda =vx_{0}$. Based on the constraints in problem (2), $\frac{v}{\theta }X\lambda =vx_{0}=1=\hat{v}{\hat{x}}_{0}$, where the last equality arises from the projection of problem (2) onto the frontier. Given that ${\hat{x}}_{0}=X\lambda$, it follows then that $\hat{v}=\frac{v}{\theta }$. [14] show that the marginal rate of substitution (MRS) is the ratio of input multipliers, that is for input *k* and *j* and *k* ≠ *j*, ${MRS}_{kj}=\frac{v_{k}}{v_{j}}$. For the projection onto the frontier, MRSkj=v^kv^j=vkθvjθ=vkvj. While the MRS between two inputs may be constant for this projection, the estimation of measures such as elasticities, that use the marginal rates, are dependent upon the levels of input use (as well as factor prices in the cost efficiency DEA framework) and will differ when projected onto the frontier.

The approach adopted here is similar to that proposed by [18] for derivation of marginal rates of substitution. We build on their approach by developing methods to estimate Hicksian and Morishima elasticities of substitution using the DEA problems (1) and (3). The approach allows for these measures to be estimated for inefficient firms. For the technical efficiency problem given by (1), the derivations arise from the relationship between the optimal solution to the technical efficiency problem given by (1) for an inefficient firm and the associated optimal solution for the same problem solved using the composite inputs and outputs projected onto the frontier [33]. For the cost efficiency problem given by (3), the objective is to minimize cost by determining the cost minimizing level of input. The optimal solution to this problem provides the information needed to estimate the Hicksian and Morishima elasticities of substitution directly.

Use of the projection onto the frontier has the added benefit of using the estimates from the DEA problem without the need to estimate directional derivatives, providing unique estimates of the economic measures of interest along the segments of the convex hull representing the frontier. This is the approach adopted in this paper and by [32]. Thus, elasticities of substitution are estimated along the segments (facets) of the frontier at the points closest to each inefficient firm. Under the assumption of constant returns to scale (CRS) the formulas for the Hicksian and Morishima elasticities of substitution derived here are unaffected, as the convexity condition in the technical and cost efficiency problems given by problems (1) and (3) does not enter the derivation of the elasticities. On the other hand, it will likely be the case that the magnitudes of the elasticities derived will likely differ when assuming CRS instead of VRS.

### The Hicksian elasticity of substitution for technical efficiency

Using the primal and dual input-oriented technical efficiency problems given by (1) and (2), the Hicksian elasticity of substitution can be derived using the Lagrangian function associated with the primal problem (1). Consider a linearly homogenous, two-input production function, *y* = *f*(*x*_1_, *x*_2_). For such a function, [2] defined the elasticity of input substitution as:

$$\sigma_{i,j}^{H}\equiv \frac{d(x_{2}/x_{1})}{d(f_{1}/f_{2})}\frac{f_{1}/f_{2}}{x_{2}/x_{1}}$$ (5)

commonly presented in logarithmic form as,

$$\sigma_{i,j}^{H}\equiv \frac{\text{dln}\left(x_{2}/x_{1}\right)}{dln(f_{1}/f_{2})}.$$ (6)

The Hicksian elasticity is equal to the rate of change in the ratio of inputs divided by the rate of change of the marginal rate of substitution between the inputs (i.e. *f*_1_/*f*_2_ represents the marginal rate of substitution of *x*_2_ for *x*_1_).

The Hicksian elasticity of substitution between two inputs *i* and *j* for an inefficient firm projected onto the production frontier (i.e. for a composite firm) evaluated using the technical efficiency problem given by (1) is given by:

σi,jH=dln(x2/x1)dln(f1/f2)=[∂(λ′xjλ′xi)∂(fifj)][(fifj)(λ′xjλ′xi)]=[(vi(λ′xi)−vj(λ′xj))(λ′xk−θoxi,o)(λ′xk−θoxj,o)(λ′xiλ′xj)(vj(λ′xk−θoxj,o)−vi(λ′xk−θoxi,o))] (7)

The full derivation of the elasticity is provided in S1 File. This elasticity shows the substitution that an inefficient DMU (at optimality) can make to inputs and remain on the production frontier and allow them to remain technically efficient. For efficient DMUs on the frontier (existing at vertex points that define the frontier), continuous derivatives do not exist, and the elasticity shown in (7) cannot be derived using traditional methods, as discussed above.

### The Hicksian elasticity of substitution for cost efficiency

Hicks’ elasticity of substitution for the cost minimization problem is analogous to the production frontier problem. For a two-input cost function, defined as *C* = *C*(*w*, *z*), the Hicksian elasticity of substitution between two inputs *($\sigma_{i,j}^{HC}$)* can be expressed in logarithmic form as:

σi,jHC=dln(wjwi)dln(CiCj)=dln(zjzi)dln(wiwj), (8)

where *w* is defined as the price (or cost) of input *z*, and *C*_*i*_ = *dc* / *dw*_*i*_ = *z*_*i*_ [36]. Thus, $\sigma_{i,j}^{HC}$ is equal to the logarithmic ratio of input quantities to input prices. Using this result, $\sigma_{i,j}^{HC}$ can be derived from the cost efficiency problem, (3). The Hicksian elasticity of substitution for cost efficiency is given by:

σi,jHC=∂ln(zjzi)∂ln(wiwj)=∂lnzj−∂lnzi∂lnwi−∂lnwj=(zj(wj−vj)wizi−zj(wj−vj)wjzj)−1−(zi(wi−vi)wizi−zi(wi−vi)wjzj)−1 (9)

where *v*_*k*_ is the shadow price on the *k*^th^ input constraint in the cost efficiency problem (3). The full derivation of the elasticity is provided in the S1 File. This elasticity measures the degree of substitutability an inefficient firm (at optimality) can make to its inputs and remain on the cost efficient frontier.

### The Morishima elasticity of substitution for cost efficiency

The Morishima formulation for the cost problem provides a more easily intuitive measurement of elasticity. Following [1], the Morishima elasticity of substitution for cost efficiency is equal to the natural log of the ratio of the *i*^*th*^ and *j*^*th*^ input prices divided by the natural log of the *j*^*th*^ input price. This elasticity can be estimated from the cost efficiency model as:

σi,jMC=dln(zizj)dln(wj)=dlnzi−dlnzjdlnwj=wjzj(wi−vi)zi−wjzj(wj−vj)zj, (10)

where *v*_*k*_ is the shadow price on the *k*^th^ input constraint in the cost efficiency problem (3). Eq (10) is derived using the cost minimizing level of inputs. The full derivation of the elasticity is provided in the S1 File. The Morishima elasticity of substitution for cost efficiency shows the change in all other inputs from a change in input price. Because it has the ability to generalize and retain most of the features of the Hicksian model, it is valuable to estimate alongside the Hicksian elasticity. In the case of more than two inputs however, the property of symmetry may not hold for Morishima elasticities [8].

## Empirical illustration

For an empirical application illustrating the elasticity measures derived above, the efficiency of Kansas non-irrigated corn operations under different tillage regimes (i.e. no-tillage, reduced-tillage, and conventional tillage) was examined. Enterprise-level data on 119 farms for the year 2014 was obtained from the Kansas Farm Management Association.

The KFMA data includes input expenses for fuel, fertilizer, herbicide, seed, labor (including both hired and unpaid labor expenses), machinery (including machinery rental and repair expenses) and input quantity of land (total acres). Following [36], an implicit quantity index for the input expense variables was constructed, with total input expenses divided by the average input cost per acre. This was done to proxy for input quantities, as only costs per input are observed in the KFMA dataset. These quantity indices serve as inputs for the input-oriented technical efficiency model. Output was measured as total value of non-irrigated corn produced. The output variable was not transformed, since corn price was assumed to remain constant across firms for the period being examined. Deriving an implicit quantity index for output would result in a scaled version of the total value of non-irrigated corn production, with the relative differences between farms remaining the same. In DEA analysis, efficiency scores are invariant to the units of measurement for inputs and outputs as long as all firms are measured in the same units, which is the case in this study [33]. Thus, transforming the output using output price should yield the same results as if no transformation was used. Table 1 contains input and output prices as well as mean, minimum, and maximum values and standard deviation of the implicit quantity indices across the 119 farms in the sample.

**Table 1:** Price and expenditure data in U.S. dollars for the 119 sample corn farms.

	Input Data							Output Data
	Fuel	Fertilizer	Herbicide	Seed	Labor	Machinery	Land	Total Corn Value
Price $/acre ($/ha)	18.75 (46.33)	86.39 (213.47)	35.74 (88.31)	64.64 (159.72)	12.56 (31.04)	113 (279.22)	28.22 (69.73)	376.42 (930.13)
Mean	498.36	498.48	498.99	496.53	2019.73	157.92	496.37	591.88
Min	12.59	4.42	7.73	4.47	180.29	2.26	8	42.40
Max	3694.19	2946.58	2620.23	2427.88	2499.65	2157.52	3123.10	750.62
Std. Dev.	3681.60	2942.16	2612.50	2423.41	2319.36	2155.26	3115.10	708.22

Note: Input and output means, minimum and maximum values, and standard deviations are quantity/price across 119 farms, with input price constant across the sample. Source of data is: Kansas Farm Management Association (http://www.agmanager.info/kfma)

Technical and cost efficiency models were estimated using models (1) and (3) for each farm using the General Algebraic Modeling System (GAMS). The results from GAMS were used to compute Hicksian and Morishima elasticities of substitution in MATLAB for each farm using Eqs (7), (9), and (10). Elasticity estimates are presented in Tables 2–4. These estimates are averages of the individual elasticity estimates across the sample of farms. The number in parentheses below the average represents the spread of the elasticity estimates across the farms examined. The first number represents the elasticity value at the 5 percent quantile, while the second number represents the elasticity value at the 95 percent quantile.

**Table 2:** Mean estimates of the Hicksian elasticity of substitution (production frontier).

	Fuel	Fertilizer	Herbicide	Seed	Labor	Machinery	Land
Fuel	-	0.38 (-0.0047, 1.61)	0.50 (-0.012, 1.77)	0.41 (-0.0040, 1.11)	0.56 (-0.018, 1.72)	0.051 (-1.86, 0.94)	0.27 (-0.0036, 0.93)
Fertilizer	-	-	0.55 (-0.012, 1.55)	0.28 (-0.0028, 1.21)	0.66 (-0.010, 1.70)	-0.0035 (-2.24, 1.02)	0.24 (-0.0054, 0.81)
Herbicide	-	-	-	0.47 (-0.0024, 1.30)	0.63 (-0.0095, 2.34)	0.15 (-2.47, 2.14)	0.49 (-0.0016, 1.54)
Seed	-	-	-	-	0.52 (-0.094, 1.47)	-0.31 (-2.56, 0.76)	0.14 (-0.041, 0.69)
Labor	-	-	-	-	-	-2.44 (-14.64, 0.85)	-0.1303 (-3.72, 0.99)
Machinery	-	-	-	-	-	-	0.37 (-0.0098, 1.087)

Note: Numbers in parentheses represent the elasticity estimate at the 5% and 95% quantile of the distribution of elasticity estimates across inefficient firms in the sample.

**Table 3:** Mean estimates of the Hicksian elasticity of substitution (cost frontier).

	Fuel	Fertilizer	Herbicide	Seed	Labor	Machinery	Land
Fuel	-	0.0021 (-0.42, 0.30)	-0.094 (-0.32, 0.066)	-0.20 (-0.96, 0.38)	-0.039 (-0.046, -0.035)	0.0079 (-0.37, 0.15)	-0.033 (-0.034, -0.031)
Fertilizer	-	-	-0.0062 (-0.13, 0.0061)	0.00063 (-0.011, 0.045)	-0.00011 (-0.0081, 0.028)	-0.014 (-0.021, -0.0076)	-0.042 (-0.049, -0.035)
Herbicide	-	-	-	-0.021 (-0.16, 0.094)	-0.0057 (-0.022, 0.023)	-0.048 (-0.21, 0.085)	-0.035 (-0.036, -0.035)
Seed	-	-	-	-	0.021 (0.0094, 0.027)	-0.0020 (-0.0069, 0.0064)	-0.035 (-0.036, -0.035)
Labor	-	-	-	-	-	0.0063 (-0.0034, 0.017)	-0.033 (-0.034, -0.033)
Machinery	-	-	-	-	-	-	-0.038 (-0.038, -0.037)

Note: Numbers in parentheses represent the elasticity estimate at the 5% and 95% quantile of the distribution of elasticity estimates across inefficient firms in the sample.

**Table 4:** Mean estimates of the Morishima elasticity of substitution (cost frontier).

	Fuel	Fertilizer	Herbicide	Seed	Labor	Machinery	Land
Fuel	-	0.015 (-0.0021, 0.058)	0.021 (-0.0095, 0.13)	0.057 (0.029, 0.11)	0.11 (0.047, 0.23)	0.028 (0.0028, 0.079)	-0.030 (-0.033, -0.025)
Fertilizer	-0.013 (-0.043, 0.012)	-	-0.0047 (-0.018, 0.0016)	0.034 (-0.0035, 0.070)	0.055 (-0.044, 0.15)	0.015 (-0.0060, 0.030)	-0.032 (-0.034, -0.030)
Herbicide	-0.0054 (-0.042, 0.027)	0.0039 (-0.00070, 0.021)	-	.0.044 (0.0057, 0.091)	0.082 (-0.022, 0.20)	0.019 (-0.0013, 0.040)	-0.031 (-0.033, -0.029)
Seed	-0.039 (-0.045, -0.034)	-0.0059 (-0.0094, 0.0033)	-0.017 (-0.023, -0.0077)	-	-0.033 (-0.039, -0.023)	-0.0015 (-0.0053, 0.0019)	-0.033 (-0.034, -0.033)
Labor	-0.029 (-0.039, -0.021)	-0.0012 (-0.0077, 0.017)	-0.0091 (-0.020, 0.011)	0.013 (0.0066, 0.016)	-	0.0048 (-0.0032, 0.012)	-0.033 (-0.034, -0.032)
Machinery	-0.0341 (-0.046, -0.012)	-0.0031 (-0.0089, 0.022)	-0.015 (-0.022, 0.0040)	0.0047 (-0.0029, 0.022)	-0.0203 (-0.043, 0.041)	-	-0.033 (-0.0333, -0.0331)
Land	0.50 (0.14, 1.27)	0.26 (0.077, 1.042)	0.40 (0.14, 0.87)	0.66 (0.46, 1.16)	1.75 (1.10, 3.50)	0.30 (0.26, 0.33)	-

Note: Numbers in parentheses represent the elasticity estimate at the 5% and 95% quantile of the distribution of elasticity estimates across inefficient firms in the sample.

The results of the estimation of Hicksian and Morishima elasticities show limited substitutability or complementarity between inputs. The mean values indicate that, at least for this set of farms for the year 2014, the response to changes in an input’s relative marginal productivity or price does not dramatically alter the proportion of inputs applied. The spread of elasticity estimates (numbers in parentheses) is more pronounced than the mean values, indicating a range of input substitutability across the farms examined. Such variation between farms may be due to a number of factors such as farm size, environmental factors, tillage methods, or management practices. Similarly, the elasticity results for the production and cost models vary. The Hicksian elasticities of substitution for technical efficiency indicate that the majority of inputs act as complements, while the Hicksian elasticity for cost efficiency indicate that the majority of inputs are substitutes (Tables 2 and 3). The Morishima elasticity for cost efficiency indicates that some inputs act as substitutes, while others act as complements. The limited substitutability of inputs is consistent with previous work on the estimation of elasticities of substitution in agricultural production [37–38].

**Fig 1:**
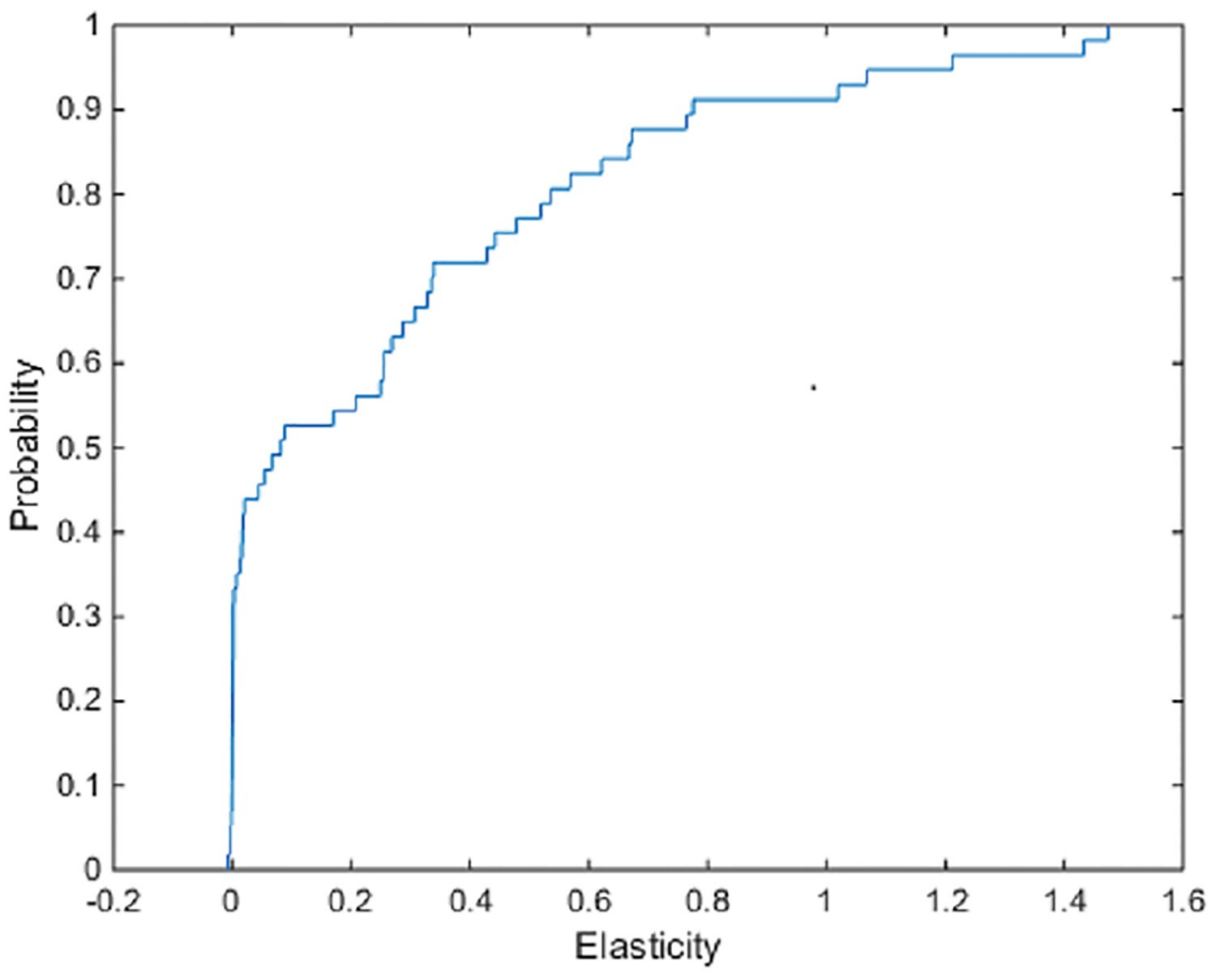
ECDF of the Hicksian production frontier elasticities of fertilizer for seed.

The results of the estimation of the Hicksian elasticities for the production frontier (Table 2) point to the set of inputs acting mostly as complements, with mean values for the elasticities ranging from -2.44 to 0.66 in magnitude (the majority of negative elasticities involve substitutions of inputs paired with machinery or land). The estimates of individual elasticity pairs vary across farms—that is, for some farms many of the inputs behave as substitutes. For example, the Hicksian elasticity of substitution for technical efficiency of fertilizer for seed has a mean value of 0.28, with a value of -0.0028 at the 5% quantile and a value of 1.21 at the 95% quantile of the empirical distribution of estimates. Fig 1 shows the estimated empirical cumulative distribution function (ecdf) of the Hicksian production frontier elasticity of fertilizer for seed. This indicates that for a majority of farms, a relative increase in the marginal product of fertilizer leads to lower application rates of seed (fertilizer and seed act as complements).

**Fig 2:**
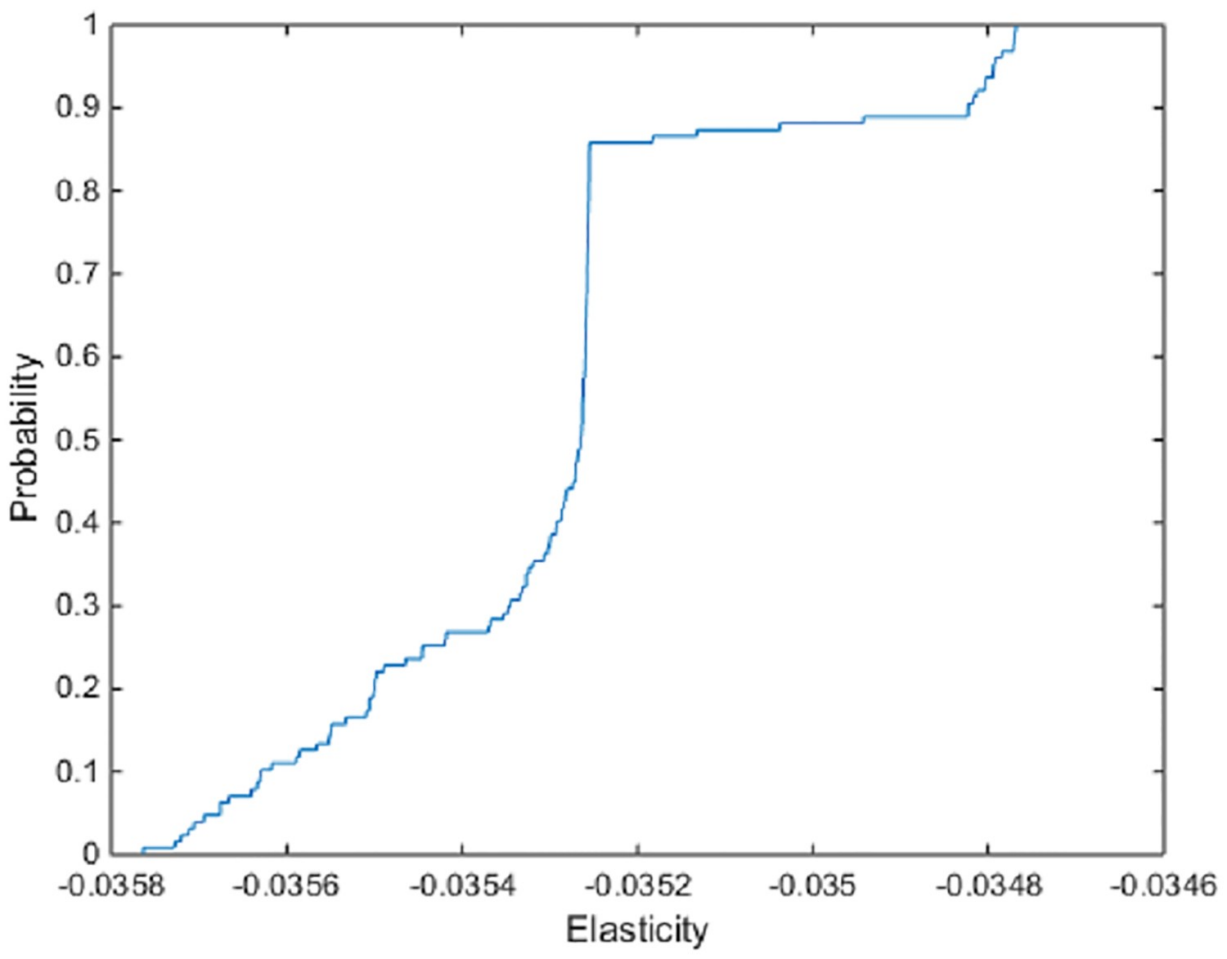
ECDF of the Hicksian cost frontier elasticities of seed for land.

The majority of Hicksian elasticity of substitution for cost efficiency estimates are smaller than their technical efficiency counterparts (Table 3) with most of the input pairs behaving as substitutes. In addition, many of the mean estimates are close to 0, suggesting that increases in the ratio of input costs does not have a strong impact on input substitutability. For example, the Hicksian cost efficiency elasticity of substitution between seed and land has a mean value of -0.035. The values at the 5% and 95% quantiles of the distribution are -0.036 and -0.035, indicating that most farms substitute land for seed given an increase in the price of seed. Fig 2 shows the estimated ecdf of the Hicksian cost frontier elasticity of seed for land across farms.

**Fig 3:**
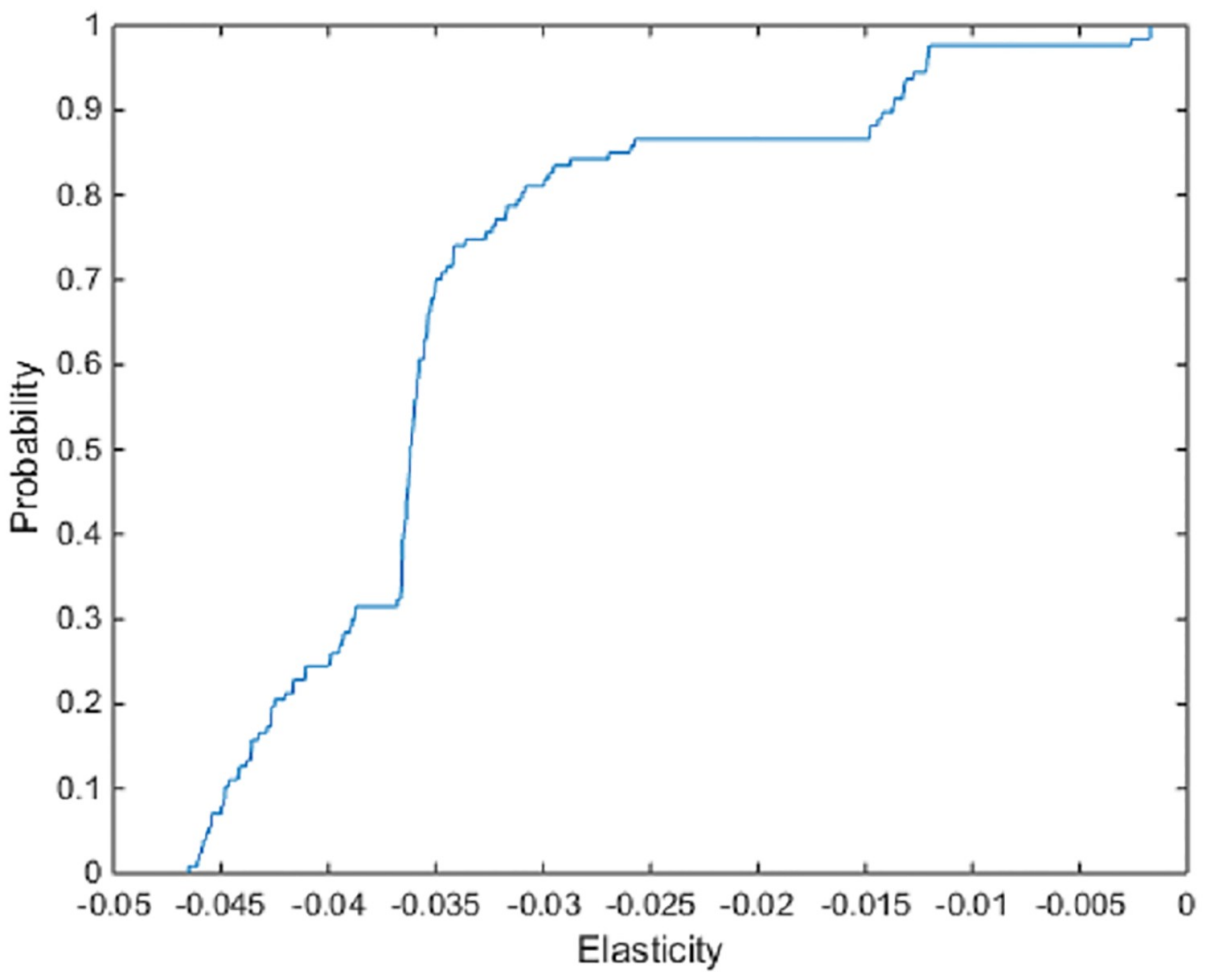
ECDF of the Morishima cost frontier elasticities of machinery for fuel.

**Fig 4:**
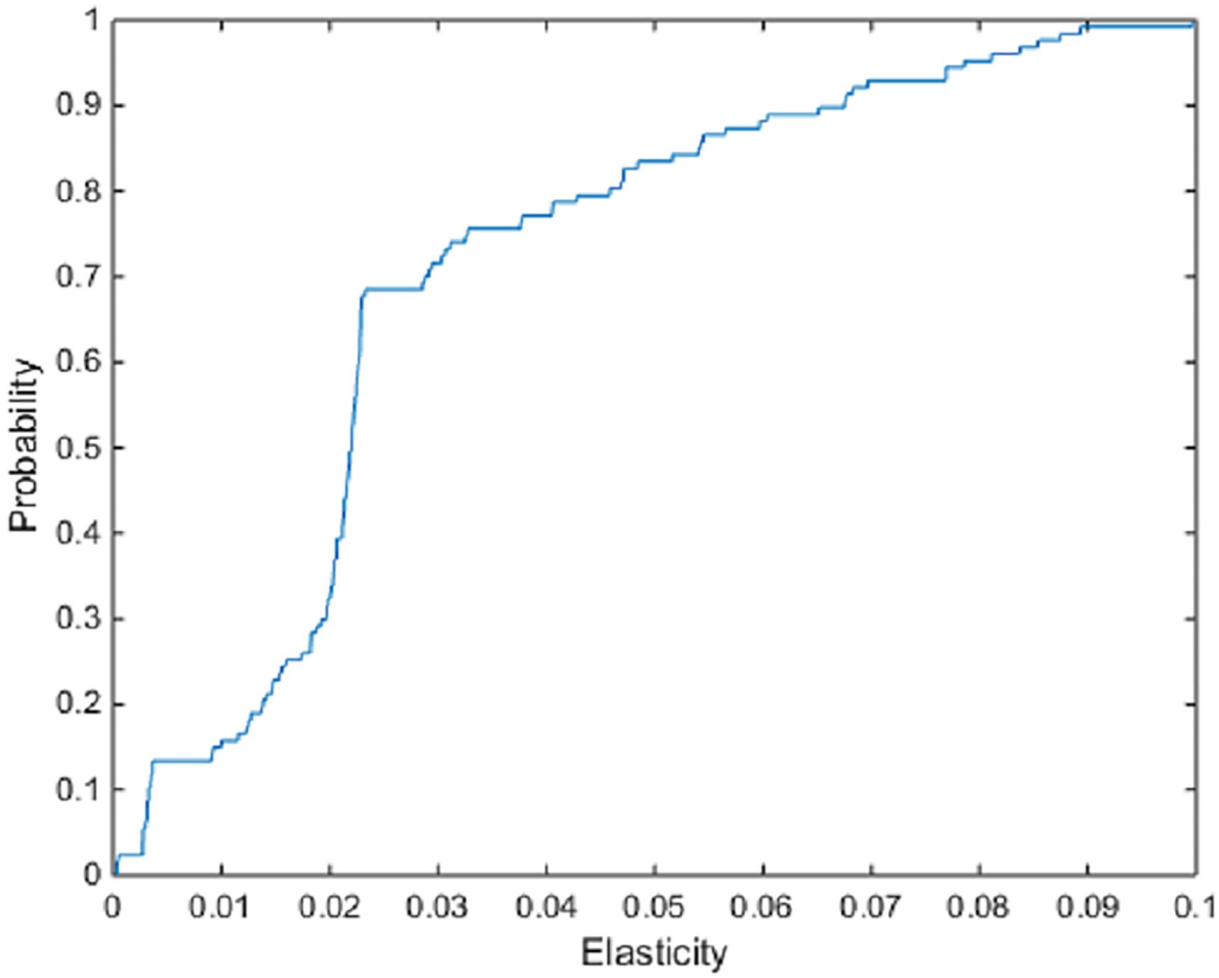
ECDF of the Morishima cost frontier elasticities of fuel for machinery.

The Morishima cost efficiency elasticity estimates are provided in Table 4. Unlike the other elasticities estimated, Morishima elasticities are not symmetric and the degree of substitutability (complementarity) between inputs is affected by direction of substitution (complementation). The results from the estimation of these relationship shows an almost equal division between input pairs that act as complements and input pairs that act as substitutes. In addition, for many of the Morishima cost efficiency elasticities and associated spreads of individual estimates are tighter. The elasticity estimates involving the substitution of machinery for fuel has a mean of -0.034, with estimates at the 5% and 95% quantiles of the empirical distribution of estimates across firms of -0.046 and -0.012, signaling slight substitutability. The elasticity involving the substitution of fuel for machinery however shows slight complementarity, with a mean of 0.028, with estimates at the 5% and 95% quantiles of the empirical distribution of estimates across firms of 0.0028 and 0.079 (Figs 3 and 4). The difference in the pair of elasticities highlight the non-symmetric aspect of the Morishima elasticities. Changes to the price of one input may have a different effect on the ease of substitutability than changes to the price of the other input. A substitution towards a particular input may therefore not be the same or have the same effect as a substitution away from that input.

The results demonstrate that the particular DEA method used to estimate the elasticities has a significant impact on the extent of an input’s substitutability. In general, the elasticities derived from the production frontier were larger than the elasticities derived from the cost frontier. The numerical implications reveal a unique point of divergence between cost frontier elasticities and production frontier elasticities. Changes in the cost frontier account for changes in input price and productivity, whereas changes in the production frontier only account for changes in input productivity. This implies that changes in the ratio of input prices may have a greater effect on the substitutability of inputs than do changes in the ratio of marginal products of two inputs.

## Conclusion

In agriculture input allocation is not fixed, but varies across time and location. Part of this variability is due to lack of knowledge on the effects of changes in input mix on the quantity produced or the cost of production. It is therefore useful to know the ease with which one input can be substituted for another. The elasticity of input substitution provides information regarding the substitutability of inputs given a change in the marginal productivity or price of an input. These elasticities have been traditionally obtained from parametric estimates of production and cost functions. Another alternative nonparametric technique for estimating these is DEA. This article develops procedures to estimate Hicksian production and cost elasticities, as well as Morishima cost elasticities of substitution. Elasticities are estimated for the composite firm on the frontier, associated with each inefficient firm, to provide useful estimates and avoid problems from deriving these measures for efficient firms at “kink” points along the frontier. The derivation of these elasticities expands the usefulness of DEA as a tool in economic analysis.

An empirical example involving Kansas famers’ corn enterprises under reduced tillage is an illustration of estimating these elasticities. Using Kansas Farm Management Association data, production and cost frontier DEA models were estimated for 119 farms. Fuel, fertilizer, herbicide, seed, labor, machinery, and land were used as inputs, with total crop value used as output. Hicksian production elasticities were estimated for each of the inefficient farms. Similarly, Hicksian and Morishima cost elasticities were estimated for each of the inefficient farms. The example shows that DEA can provide elasticity estimates for a sample of DMUs and provide individual estimates for the set of DMUs along the production and cost frontiers. In providing a range of elasticity estimates, DEA can help farmers manage their inputs by examining the different conditions under which two inputs are classified as substitutes and as complements. In addition, the stability of these measures across time can be determined.

A particular area of interest for future research is the estimation of output supply elasticities. Output supply elasticities show the response of a DMU in terms of output, given a change in the output price. Using the output-oriented BCC model, one could estimate how a change to price effects the production decisions of the DMU. As with the estimation of elasticities of input substitution, this is a worthwhile line of inquiry from a farm management perspective. In reality, farmers consider the price of output as well as the price or marginal productivity of inputs when making judgments regarding the application of farm inputs. Such a study would complement this one, giving a more holistic and complete depiction of producer decision-making. Beyond this, other future research can extend the approach examined in this paper to more advanced areas in DEA, including elasticities of transformation for multiple outputs or outcomes [39]; examination of firm intensification [40]; examination of efficiency dynamics and productivity over time [41]; derivation using advanced methods such as fuzzy DEA [42]; and incorporation into conditional DEA approaches [43].

## Supporting information

S1 File Elasticity derivations. (DOCX)

S2 File DEA data. (CSV)
