# Supplementary material for: Economic elasticities of input substitution using data envelopment analysis
Source: PLoS One. 2019 Aug 8;14(8):e0220478. doi: 10.1371/journal.pone.0220478 (PMC6687138; doi:10.1371/journal.pone.0220478)
Supplement: S1 File — (DOCX) [file pone.0220478.s001.docx]

**S1 File. Elasticity derivations**

**A. Derivation of the elasticity of substitution for inefficient firms (production problem)**

**A.1. The Hicksian elasticity of substitution**

The corresponding (primal) technical efficiency problem is given by:

| *BCC(min):* | ${}_{\theta_{o},\lambda}^{min}{\theta_{o}}$ |  |  |
| --- | --- | --- | --- |
| *Subject to:* | $\boldsymbol{\lambda}^{\boldsymbol{'}}\boldsymbol{x}_{\boldsymbol{k}}\leq\theta_{o}x_{k,o} \forall\text{k inputs}$ | $\to\boldsymbol{v}$ |  |
|  | $\boldsymbol{\lambda}^{\boldsymbol{'}}\boldsymbol{y}_{\boldsymbol{m}}\geq y_{m,o}$ $\boldsymbol{\forall}\text{m outputs}$ | $\to\boldsymbol{u}$ |  |
|  | $\boldsymbol{e}^{\boldsymbol{'}}\boldsymbol{\lambda}=1$ | ${\to u}_{0}$ |  |
|  | $\boldsymbol{\lambda}\geq0$ |  |  |

Let $L$ denote the Langrangian function for the technical efficiency problem, where $\theta_{o}$ refers to the objective value for firm 0, the firm of interest.

$$L=\theta_{o}+\sum_{k} v_{k}\left( \boldsymbol{\lambda}^{\boldsymbol{'}}\boldsymbol{x}_{\boldsymbol{k}}-\theta_{o}x_{k,o} \right)-\sum_{m} u_{m}\left( \boldsymbol{\lambda}^{\boldsymbol{'}}\boldsymbol{y}_{\boldsymbol{m}}-y_{m,o} \right)+u_{o}\left( \boldsymbol{e}^{\boldsymbol{'}}\boldsymbol{\lambda}-1 \right).$$

Let $\hat{x}_{k}=\boldsymbol{\lambda}^{\boldsymbol{'}}\boldsymbol{x}_{\boldsymbol{k}}$ be the *k*^th^ composite input for the firm of interest [18]. The Hicksian elasticity of input substitution of interest for an inefficient firm can be represented by:

$\sigma_{i,j}^{H}=\left[ \frac{\partial\left( \frac{\hat{x}_{j}}{\hat{x}_{i}} \right)}{\partial\left( \frac{f_{i}}{f_{j}} \right)} \right]\left[ \frac{\left( \frac{f_{i}}{f_{j}} \right)}{\left( \frac{\hat{x}_{j}}{\hat{x}_{i}} \right)} \right]=\left[ \frac{\partial\left( \frac{\boldsymbol{\lambda}^{\boldsymbol{'}}\boldsymbol{x}_{\boldsymbol{j}}}{\boldsymbol{\lambda}^{\boldsymbol{'}}\boldsymbol{x}_{\boldsymbol{i}}} \right)}{\partial\left( \frac{f_{i}}{f_{j}} \right)} \right]\left[ \frac{\left( \frac{f_{i}}{f_{j}} \right)}{\left( \frac{\boldsymbol{\lambda}^{\boldsymbol{'}}\boldsymbol{x}_{\boldsymbol{j}}}{\boldsymbol{\lambda}^{\boldsymbol{'}}\boldsymbol{x}_{\boldsymbol{i}}} \right)} \right]$,

where the elasticity measure is derived with respect to the composite inputs. Following [18] and the arguments in the paper, the marginal rate of technical substitution (MRTS) equals $\frac{f_{i}}{f_{j}}=\frac{\hat{v}_{i}}{\hat{v}_{j}}=\frac{v_{i}}{v_{j}}$, where $\hat{v}_{k}$ is the shadow price on the input constraint in the technical efficiency problem above when projected onto the frontier. Given this relationship and that the composite inputs are estimated within the technical efficiency problem above, we can utilize the *BCC* DEA technical efficiency problem to estimate the Hicksian elasticities of substitution. Substituting in the MRTS,

$\sigma_{i,j}^{H}=\left[ \frac{\partial\left( \frac{\boldsymbol{\lambda}^{\boldsymbol{'}}\boldsymbol{x}_{\boldsymbol{j}}}{\boldsymbol{\lambda}^{\boldsymbol{'}}\boldsymbol{x}_{\boldsymbol{i}}} \right)}{\partial\left( \frac{v_{i}}{v_{j}} \right)} \right]\left[ \frac{\left( \frac{v_{i}}{v_{j}} \right)}{\left( \frac{\boldsymbol{\lambda}^{\boldsymbol{'}}\boldsymbol{x}_{\boldsymbol{j}}}{\boldsymbol{\lambda}^{\boldsymbol{'}}\boldsymbol{x}_{\boldsymbol{i}}} \right)} \right]$.

The first component of the Hicksian elasticity can be derived as (using the quotient rule):

$$\frac{\partial\left( \frac{\boldsymbol{\lambda}^{\boldsymbol{'}}\boldsymbol{x}_{\boldsymbol{j}}}{\boldsymbol{\lambda}^{\boldsymbol{'}}\boldsymbol{x}_{\boldsymbol{i}}} \right)}{\partial\left( \frac{v_{i}}{v_{j}} \right)}=\left[ \frac{\left( \frac{\boldsymbol{\lambda}^{\boldsymbol{'}}\boldsymbol{x}_{\boldsymbol{i}}\partial\boldsymbol{\lambda}^{\boldsymbol{'}}\boldsymbol{x}_{\boldsymbol{j}}-\boldsymbol{\lambda}^{\boldsymbol{'}}\boldsymbol{x}_{\boldsymbol{j}}\partial\boldsymbol{\lambda}^{\boldsymbol{'}}\boldsymbol{x}_{\boldsymbol{i}}}{{\boldsymbol{\lambda}^{\boldsymbol{'}}\boldsymbol{x}}_{i}^{2}} \right)}{\left( \frac{v_{j}\partial v_{i}-v_{i}\partial v_{j}}{v_{j}^{2}} \right)} \right].$$

Rearranging terms and using the inverse operation gives:

$$\frac{\partial\left( \frac{\boldsymbol{\lambda}^{\boldsymbol{'}}\boldsymbol{x}_{\boldsymbol{j}}}{\boldsymbol{\lambda}^{\boldsymbol{'}}\boldsymbol{x}_{\boldsymbol{i}}} \right)}{\partial\left( \frac{v_{i}}{v_{j}} \right)}=\left[ \frac{v_{j}^{2}}{\left( \boldsymbol{\lambda}^{\boldsymbol{'}}\boldsymbol{x}_{\boldsymbol{i}} \right)^{2}} \right]\left[ \frac{\boldsymbol{\lambda}^{\boldsymbol{'}}\boldsymbol{x}_{\boldsymbol{i}}\partial\boldsymbol{\lambda}^{\boldsymbol{'}}\boldsymbol{x}_{\boldsymbol{j}}-\boldsymbol{\lambda}^{\boldsymbol{'}}\boldsymbol{x}_{\boldsymbol{j}}\partial\boldsymbol{\lambda}^{\boldsymbol{'}}\boldsymbol{x}_{\boldsymbol{i}}}{v_{j}\partial v_{i}-v_{i}\partial v_{j}} \right]=\left[ \frac{v_{j}^{2}}{\left( \boldsymbol{\lambda}^{\boldsymbol{'}}\boldsymbol{x}_{\boldsymbol{i}} \right)^{2}} \right]\left[ \frac{v_{j}\partial v_{i}-v_{i}\partial v_{j}}{\boldsymbol{\lambda}^{\boldsymbol{'}}\boldsymbol{x}_{\boldsymbol{i}}\partial\boldsymbol{\lambda}^{\boldsymbol{'}}\boldsymbol{x}_{\boldsymbol{j}}-\boldsymbol{\lambda}^{\boldsymbol{'}}\boldsymbol{x}_{\boldsymbol{j}}\partial\boldsymbol{\lambda}^{\boldsymbol{'}}\boldsymbol{x}_{\boldsymbol{i}}} \right]^{-1}=\left[ \frac{v_{j}^{2}}{\left( \boldsymbol{\lambda}^{\boldsymbol{'}}\boldsymbol{x}_{\boldsymbol{i}} \right)^{2}} \right]\left[ \frac{v_{j}\partial v_{i}}{\boldsymbol{\lambda}^{\boldsymbol{'}}\boldsymbol{x}_{\boldsymbol{i}}\partial\boldsymbol{\lambda}^{\boldsymbol{'}}\boldsymbol{x}_{\boldsymbol{j}}-\boldsymbol{\lambda}^{\boldsymbol{'}}\boldsymbol{x}_{\boldsymbol{j}}\partial\boldsymbol{\lambda}^{\boldsymbol{'}}\boldsymbol{x}_{\boldsymbol{i}}}-\frac{v_{i}\partial v_{j}}{\boldsymbol{\lambda}^{\boldsymbol{'}}\boldsymbol{x}_{\boldsymbol{i}}\partial\boldsymbol{\lambda}^{\boldsymbol{'}}\boldsymbol{x}_{\boldsymbol{j}}-\boldsymbol{\lambda}^{\boldsymbol{'}}\boldsymbol{x}_{\boldsymbol{j}}\partial\boldsymbol{\lambda}^{\boldsymbol{'}}\boldsymbol{x}_{\boldsymbol{i}}} \right]^{-1}$$

$$=\left[ \frac{v_{j}^{2}}{\left( \boldsymbol{\lambda}^{\boldsymbol{'}}\boldsymbol{x}_{\boldsymbol{i}} \right)^{2}} \right]\left[ \left( \frac{\boldsymbol{\lambda}^{\boldsymbol{'}}\boldsymbol{x}_{\boldsymbol{i}}\partial\boldsymbol{\lambda}^{\boldsymbol{'}}\boldsymbol{x}_{\boldsymbol{j}}-\boldsymbol{\lambda}^{\boldsymbol{'}}\boldsymbol{x}_{\boldsymbol{j}}\partial\boldsymbol{\lambda}^{\boldsymbol{'}}\boldsymbol{x}_{\boldsymbol{i}}}{v_{j}\partial v_{i}} \right)^{-1}-\left( \frac{\boldsymbol{\lambda}^{\boldsymbol{'}}\boldsymbol{x}_{\boldsymbol{i}}\partial\boldsymbol{\lambda}^{\boldsymbol{'}}\boldsymbol{x}_{\boldsymbol{j}}-\boldsymbol{\lambda}^{\boldsymbol{'}}\boldsymbol{x}_{\boldsymbol{j}}\partial\boldsymbol{\lambda}^{\boldsymbol{'}}\boldsymbol{x}_{\boldsymbol{i}}}{v_{i}\partial v_{j}} \right)^{-1} \right]^{-1}$$

$$=\left[ \frac{v_{j}^{2}}{\left( \boldsymbol{\lambda}^{\boldsymbol{'}}\boldsymbol{x}_{\boldsymbol{i}} \right)^{2}} \right]\left[ \left( \left[ \frac{v_{j}\partial v_{i}}{\boldsymbol{\lambda}^{\boldsymbol{'}}\boldsymbol{x}_{\boldsymbol{i}}\partial\boldsymbol{\lambda}^{\boldsymbol{'}}\boldsymbol{x}_{\boldsymbol{j}}} \right]^{-1}-\left[ \frac{v_{j}\partial v_{i}}{\boldsymbol{\lambda}^{\boldsymbol{'}}\boldsymbol{x}_{\boldsymbol{j}}\partial\boldsymbol{\lambda}^{\boldsymbol{'}}\boldsymbol{x}_{\boldsymbol{i}}} \right]^{-1} \right)^{-1}-\left( \left[ \frac{v_{i}\partial v_{j}}{\boldsymbol{\lambda}^{\boldsymbol{'}}\boldsymbol{x}_{\boldsymbol{i}}\partial\boldsymbol{\lambda}^{\boldsymbol{'}}\boldsymbol{x}_{\boldsymbol{j}}} \right]^{-1}-\left[ \frac{v_{i}\partial v_{j}}{\boldsymbol{\lambda}^{\boldsymbol{'}}\boldsymbol{x}_{\boldsymbol{j}}\partial\boldsymbol{\lambda}^{\boldsymbol{'}}\boldsymbol{x}_{\boldsymbol{i}}} \right]^{-1} \right)^{-1} \right]^{-1}.$$

Some of the individual components from the above derivation can be represented as:

(i)$\left[ \frac{v_{j}{\partial v}_{i}}{\boldsymbol{\lambda}^{\boldsymbol{'}}\boldsymbol{x}_{\boldsymbol{i}}\partial\boldsymbol{\lambda}^{\boldsymbol{'}}\boldsymbol{x}_{\boldsymbol{j}}} \right]=\left[ \left( \frac{\partial L}{\partial L} \right)\left( \frac{v_{j}{\partial v}_{i}}{\boldsymbol{\lambda}^{\boldsymbol{'}}\boldsymbol{x}_{\boldsymbol{i}}\partial\boldsymbol{\lambda}^{\boldsymbol{'}}\boldsymbol{x}_{\boldsymbol{j}}} \right) \right]=\left[ \frac{\left( v_{j} \right)\left( \frac{\partial L}{\partial\boldsymbol{\lambda}^{\boldsymbol{'}}\boldsymbol{x}_{\boldsymbol{j}}} \right)}{\left( \boldsymbol{\lambda}^{\boldsymbol{'}}\boldsymbol{x}_{\boldsymbol{i}} \right)\left( \frac{\partial L}{{\partial v}_{i}} \right)} \right]=\left[ \frac{v_{j}^{2}}{\left( \boldsymbol{\lambda}^{\boldsymbol{'}}\boldsymbol{x}_{\boldsymbol{i}} \right)\left( \frac{\partial L}{{\partial v}_{i}} \right)} \right]=\left[ \frac{v_{j}^{2}}{\left( \boldsymbol{\lambda}^{\boldsymbol{'}}\boldsymbol{x}_{\boldsymbol{i}} \right)\left( \boldsymbol{\lambda}^{\boldsymbol{'}}\boldsymbol{x}_{\boldsymbol{i}}-\theta_{o}x_{i,o} \right)} \right]$,

where $\frac{\partial L}{\partial v_{i}}=\boldsymbol{\lambda}^{\boldsymbol{'}}\boldsymbol{x}_{\boldsymbol{i}}-\theta_{o}x_{i,o}$;

(ii) $\left[ \frac{v_{j}{\partial v}_{i}}{\boldsymbol{\lambda}^{\boldsymbol{'}}\boldsymbol{x}_{\boldsymbol{j}}\partial\boldsymbol{\lambda}^{\boldsymbol{'}}\boldsymbol{x}_{\boldsymbol{i}}} \right]=\left[ \left( \frac{\partial L}{\partial L} \right)\left( \frac{v_{j}{\partial v}_{i}}{\boldsymbol{\lambda}^{\boldsymbol{'}}\boldsymbol{x}_{\boldsymbol{j}}\partial\boldsymbol{\lambda}^{\boldsymbol{'}}\boldsymbol{x}_{\boldsymbol{i}}} \right) \right]=\left[ \frac{\left( v_{j} \right)\left( \frac{\partial L}{\partial\boldsymbol{\lambda}^{\boldsymbol{'}}\boldsymbol{x}_{\boldsymbol{i}}} \right)}{\left( \boldsymbol{\lambda}^{\boldsymbol{'}}\boldsymbol{x}_{\boldsymbol{j}} \right)\left( \frac{\partial L}{{\partial v}_{i}} \right)} \right]=\left[ \frac{{v_{i}v}_{j}}{\left( \boldsymbol{\lambda}^{\boldsymbol{'}}\boldsymbol{x}_{\boldsymbol{j}} \right)\left( \frac{\partial L}{{\partial v}_{i}} \right)} \right]=\left[ \frac{{v_{i}v}_{j}}{\left( \boldsymbol{\lambda}^{\boldsymbol{'}}\boldsymbol{x}_{\boldsymbol{j}} \right)\left( \boldsymbol{\lambda}^{\boldsymbol{'}}\boldsymbol{x}_{\boldsymbol{i}}-\theta_{o}x_{i,o} \right)} \right]$;

(iii) $\left[ \frac{v_{i}{\partial v}_{j}}{\boldsymbol{\lambda}^{\boldsymbol{'}}\boldsymbol{x}_{\boldsymbol{i}}\partial\boldsymbol{\lambda}^{\boldsymbol{'}}\boldsymbol{x}_{\boldsymbol{j}}} \right]=\left[ \left( \frac{\partial L}{\partial L} \right)\left( \frac{v_{i}{\partial v}_{j}}{\boldsymbol{\lambda}^{\boldsymbol{'}}\boldsymbol{x}_{\boldsymbol{i}}\partial\boldsymbol{\lambda}^{\boldsymbol{'}}\boldsymbol{x}_{\boldsymbol{j}}} \right) \right]=\left[ \frac{\left( v_{i} \right)\left( \frac{\partial L}{\partial\boldsymbol{\lambda}^{\boldsymbol{'}}\boldsymbol{x}_{\boldsymbol{j}}} \right)}{\left( \boldsymbol{\lambda}^{\boldsymbol{'}}\boldsymbol{x}_{\boldsymbol{i}} \right)\left( \frac{\partial L}{{\partial v}_{j}} \right)} \right]=\left[ \frac{{v_{i}v}_{j}}{\left( \boldsymbol{\lambda}^{\boldsymbol{'}}\boldsymbol{x}_{\boldsymbol{i}} \right)\left( \frac{\partial L}{{\partial v}_{j}} \right)} \right]=\left[ \frac{{v_{i}v}_{j}}{\left( \boldsymbol{\lambda}^{\boldsymbol{'}}\boldsymbol{x}_{\boldsymbol{i}} \right)\left( \boldsymbol{\lambda}^{\boldsymbol{'}}\boldsymbol{x}_{\boldsymbol{j}}-\theta_{o}x_{j,o} \right)} \right]$;and

(iv) $\left[ \frac{v_{i}{\partial v}_{j}}{\boldsymbol{\lambda}^{\boldsymbol{'}}\boldsymbol{x}_{\boldsymbol{j}}\partial\boldsymbol{\lambda}^{\boldsymbol{'}}\boldsymbol{x}_{\boldsymbol{i}}} \right]=\left[ \left( \frac{\partial L}{\partial L} \right)\left( \frac{v_{i}{\partial v}_{j}}{\boldsymbol{\lambda}^{\boldsymbol{'}}\boldsymbol{x}_{\boldsymbol{j}}\partial\boldsymbol{\lambda}^{\boldsymbol{'}}\boldsymbol{x}_{\boldsymbol{i}}} \right) \right]=\left[ \frac{\left( v_{i} \right)\left( \frac{\partial L}{\partial\boldsymbol{\lambda}^{\boldsymbol{'}}\boldsymbol{x}_{\boldsymbol{i}}} \right)}{\left( \boldsymbol{\lambda}^{\boldsymbol{'}}\boldsymbol{x}_{\boldsymbol{j}} \right)\left( \frac{\partial L}{{\partial v}_{j}} \right)} \right]=\left[ \frac{v_{i}^{2}}{\left( \boldsymbol{\lambda}^{\boldsymbol{'}}\boldsymbol{x}_{\boldsymbol{j}} \right)\left( \frac{\partial L}{{\partial v}_{j}} \right)} \right]=\left[ \frac{v_{i}^{2}}{\left( \boldsymbol{\lambda}^{\boldsymbol{'}}\boldsymbol{x}_{\boldsymbol{j}} \right)\left( \boldsymbol{\lambda}^{\boldsymbol{'}}\boldsymbol{x}_{\boldsymbol{j}}-\theta_{o}x_{j,o} \right)} \right]$.

Substituting (i) to (iv) back into the prior derivation gives:

$\frac{\partial\left( \frac{\boldsymbol{\lambda}^{\boldsymbol{'}}\boldsymbol{x}_{\boldsymbol{j}}}{\boldsymbol{\lambda}^{\boldsymbol{'}}\boldsymbol{x}_{\boldsymbol{i}}} \right)}{\partial\left( \frac{v_{i}}{v_{j}} \right)}=\left[ \frac{v_{j}^{2}}{\left( \boldsymbol{\lambda}^{\boldsymbol{'}}\boldsymbol{x}_{\boldsymbol{i}} \right)^{2}} \right]\left[ \left( {\left[ \frac{v_{j}^{2}}{\left( \boldsymbol{\lambda}^{\boldsymbol{'}}\boldsymbol{x}_{\boldsymbol{i}} \right)\left( \boldsymbol{\lambda}^{\boldsymbol{'}}\boldsymbol{x}_{\boldsymbol{i}}-\theta_{o}x_{i,o} \right)} \right]^{-1}-\left[ \frac{{v_{i}v}_{j}}{\left( \boldsymbol{\lambda}^{\boldsymbol{'}}\boldsymbol{x}_{\boldsymbol{j}} \right)\left( \boldsymbol{\lambda}^{\boldsymbol{'}}\boldsymbol{x}_{\boldsymbol{i}}-\theta_{o}x_{i,o} \right)} \right]}^{-1} \right)^{-1}-\left( \left[ \frac{{v_{i}v}_{j}}{\left( \boldsymbol{\lambda}^{\boldsymbol{'}}\boldsymbol{x}_{\boldsymbol{i}} \right)\left( \boldsymbol{\lambda}^{\boldsymbol{'}}\boldsymbol{x}_{\boldsymbol{j}}-\theta_{o}x_{j,o} \right)} \right]^{-1}-\left[ \frac{v_{i}^{2}}{\left( \boldsymbol{\lambda}^{\boldsymbol{'}}\boldsymbol{x}_{\boldsymbol{j}} \right)\left( \boldsymbol{\lambda}^{\boldsymbol{'}}\boldsymbol{x}_{\boldsymbol{j}}-\theta_{o}x_{j,o} \right)} \right]^{-1} \right)^{-1} \right]^{-1}$.

Now reversing the inverse operations originally used and combining terms, gives:

$$\frac{\partial\left( \frac{\boldsymbol{\lambda}^{\boldsymbol{'}}\boldsymbol{x}_{\boldsymbol{j}}}{\boldsymbol{\lambda}^{\boldsymbol{'}}\boldsymbol{x}_{\boldsymbol{i}}} \right)}{\partial\left( \frac{v_{i}}{v_{j}} \right)}=\left[ \frac{v_{j}^{2}}{\left( \boldsymbol{\lambda}^{\boldsymbol{'}}\boldsymbol{x}_{\boldsymbol{i}} \right)^{2}} \right]\left[ \left( \left[ \frac{\left( \boldsymbol{\lambda}^{\boldsymbol{'}}\boldsymbol{x}_{\boldsymbol{i}} \right)\left( \boldsymbol{\lambda}^{\boldsymbol{'}}\boldsymbol{x}_{\boldsymbol{i}}-\theta_{o}x_{i,o} \right)}{v_{j}^{2}} \right]-\left[ \frac{\left( \boldsymbol{\lambda}^{\boldsymbol{'}}\boldsymbol{x}_{\boldsymbol{j}} \right)\left( \boldsymbol{\lambda}^{\boldsymbol{'}}\boldsymbol{x}_{\boldsymbol{i}}-\theta_{o}x_{i,o} \right)}{{v_{i}v}_{j}} \right] \right)^{-1}-\left( \left[ \frac{\left( \boldsymbol{\lambda}^{\boldsymbol{'}}\boldsymbol{x}_{\boldsymbol{i}} \right)\left( \boldsymbol{\lambda}^{\boldsymbol{'}}\boldsymbol{x}_{\boldsymbol{j}}-\theta_{o}x_{j,o} \right)}{{v_{i}v}_{j}} \right]-\left[ \frac{\left( \boldsymbol{\lambda}^{\boldsymbol{'}}\boldsymbol{x}_{\boldsymbol{j}} \right)\left( \boldsymbol{\lambda}^{\boldsymbol{'}}\boldsymbol{x}_{\boldsymbol{j}}-\theta_{o}x_{j,o} \right)}{v_{i}^{2}} \right] \right)^{-1} \right]^{-1}$$

$$=\left[ \frac{v_{j}^{2}}{\left( \boldsymbol{\lambda}^{\boldsymbol{'}}\boldsymbol{x}_{\boldsymbol{i}} \right)^{2}} \right]\left[ \left( \left[ \frac{v_{i}\left( \boldsymbol{\lambda}^{\boldsymbol{'}}\boldsymbol{x}_{\boldsymbol{i}} \right)\left( \boldsymbol{\lambda}^{\boldsymbol{'}}\boldsymbol{x}_{\boldsymbol{i}}-\theta_{o}x_{i,o} \right)}{v_{i}v_{j}^{2}} \right]-\left[ \frac{v_{j}\left( \boldsymbol{\lambda}^{\boldsymbol{'}}\boldsymbol{x}_{\boldsymbol{j}} \right)\left( \boldsymbol{\lambda}^{\boldsymbol{'}}\boldsymbol{x}_{\boldsymbol{i}}-\theta_{o}x_{i,o} \right)}{v_{i}v_{j}^{2}} \right] \right)^{-1}-\left( \left[ \frac{v_{i}\left( \boldsymbol{\lambda}^{\boldsymbol{'}}\boldsymbol{x}_{\boldsymbol{i}} \right)\left( \boldsymbol{\lambda}^{\boldsymbol{'}}\boldsymbol{x}_{\boldsymbol{j}}-\theta_{o}x_{j,o} \right)}{v_{i}^{2}v_{j}} \right]-\left[ \frac{v_{j}\left( \boldsymbol{\lambda}^{\boldsymbol{'}}\boldsymbol{x}_{\boldsymbol{j}} \right)\left( \boldsymbol{\lambda}^{\boldsymbol{'}}\boldsymbol{x}_{\boldsymbol{j}}-\theta_{o}x_{j,o} \right)}{v_{i}^{2}v_{j}} \right] \right)^{-1} \right]^{-1}$$

$$=\left[ \frac{v_{j}^{2}}{\left( \boldsymbol{\lambda}^{\boldsymbol{'}}\boldsymbol{x}_{\boldsymbol{i}} \right)^{2}} \right]\left[ \left( \left[ \frac{{v_{i}\left( \boldsymbol{\lambda}^{\boldsymbol{'}}\boldsymbol{x}_{\boldsymbol{i}} \right)\left( \boldsymbol{\lambda}^{\boldsymbol{'}}\boldsymbol{x}_{\boldsymbol{i}}-\theta_{o}x_{i,o} \right)-v}_{j}\left( \boldsymbol{\lambda}^{\boldsymbol{'}}\boldsymbol{x}_{\boldsymbol{j}} \right)\left( \boldsymbol{\lambda}^{\boldsymbol{'}}\boldsymbol{x}_{\boldsymbol{i}}-\theta_{o}x_{i,o} \right)}{v_{i}v_{j}^{2}} \right] \right)^{-1}-\left( \left[ \frac{v_{i}\left( \boldsymbol{\lambda}^{\boldsymbol{'}}\boldsymbol{x}_{\boldsymbol{i}} \right)\left( \boldsymbol{\lambda}^{\boldsymbol{'}}\boldsymbol{x}_{\boldsymbol{j}}-\theta_{o}x_{j,o} \right)-v_{j}\left( \boldsymbol{\lambda}^{\boldsymbol{'}}\boldsymbol{x}_{\boldsymbol{j}} \right)\left( \boldsymbol{\lambda}^{\boldsymbol{'}}\boldsymbol{x}_{\boldsymbol{j}}-\theta_{o}x_{j,o} \right)}{v_{i}^{2}v_{j}} \right] \right)^{-1} \right]^{-1}$$

$$=\left[ \frac{v_{j}^{2}}{\left( \boldsymbol{\lambda}^{\boldsymbol{'}}\boldsymbol{x}_{\boldsymbol{i}} \right)^{2}} \right]\left[ \left( \frac{v_{i}v_{j}^{2}}{\left( v_{i}\left( \boldsymbol{\lambda}^{\boldsymbol{'}}\boldsymbol{x}_{\boldsymbol{i}} \right)-v_{j}\left( \boldsymbol{\lambda}^{\boldsymbol{'}}\boldsymbol{x}_{\boldsymbol{j}} \right) \right)\left( \boldsymbol{\lambda}^{\boldsymbol{'}}\boldsymbol{x}_{\boldsymbol{i}}-\theta_{o}x_{i,o} \right)} \right)-\left( \frac{v_{i}^{2}v_{j}}{\left( v_{i}\left( \boldsymbol{\lambda}^{\boldsymbol{'}}\boldsymbol{x}_{\boldsymbol{i}} \right)-v_{j}\left( \boldsymbol{\lambda}^{\boldsymbol{'}}\boldsymbol{x}_{\boldsymbol{j}} \right) \right)\left( \boldsymbol{\lambda}^{\boldsymbol{'}}\boldsymbol{x}_{\boldsymbol{j}}-\theta_{o}x_{j,o} \right)} \right) \right]^{-1}$$

$$=\left[ \frac{v_{j}^{2}}{\left( \boldsymbol{\lambda}^{\boldsymbol{'}}\boldsymbol{x}_{\boldsymbol{i}} \right)^{2}} \right]\left[ \frac{\left( v_{i}v_{j}^{2}\left( \boldsymbol{\lambda}^{\boldsymbol{'}}\boldsymbol{x}_{\boldsymbol{j}}-\theta_{o}x_{j,o} \right)-v_{i}^{2}v_{j}\left( \boldsymbol{\lambda}^{\boldsymbol{'}}\boldsymbol{x}_{\boldsymbol{i}}-\theta_{o}x_{i,o} \right) \right)}{\left( v_{i}\left( \boldsymbol{\lambda}^{\boldsymbol{'}}\boldsymbol{x}_{\boldsymbol{i}} \right)-v_{j}\left( \boldsymbol{\lambda}^{\boldsymbol{'}}\boldsymbol{x}_{\boldsymbol{j}} \right) \right)\left( \boldsymbol{\lambda}^{\boldsymbol{'}}\boldsymbol{x}_{\boldsymbol{i}}-\theta_{o}x_{i,o} \right)\left( \boldsymbol{\lambda}^{\boldsymbol{'}}\boldsymbol{x}_{\boldsymbol{j}}-\theta_{o}x_{j,o} \right)} \right]^{-1}$$

$$=\left[ \frac{v_{j}^{2}}{\left( \boldsymbol{\lambda}^{\boldsymbol{'}}\boldsymbol{x}_{\boldsymbol{i}} \right)^{2}} \right]\left[ \frac{\left( v_{i}\left( \boldsymbol{\lambda}^{\boldsymbol{'}}\boldsymbol{x}_{\boldsymbol{i}} \right)-v_{j}\left( \boldsymbol{\lambda}^{\boldsymbol{'}}\boldsymbol{x}_{\boldsymbol{j}} \right) \right)\left( \boldsymbol{\lambda}^{\boldsymbol{'}}\boldsymbol{x}_{\boldsymbol{i}}-\theta_{o}x_{i,o} \right)\left( \boldsymbol{\lambda}^{\boldsymbol{'}}\boldsymbol{x}_{\boldsymbol{j}}-\theta_{o}x_{j,o} \right)}{\left( v_{i}v_{j} \right)\left( v_{j}\left( \boldsymbol{\lambda}^{\boldsymbol{'}}\boldsymbol{x}_{\boldsymbol{j}}-\theta_{o}x_{j,o} \right)-v_{i}\left( \boldsymbol{\lambda}^{\boldsymbol{'}}\boldsymbol{x}_{\boldsymbol{i}}-\theta_{o}x_{i,o} \right) \right)} \right]$$

$$=\left[ \frac{v_{j}\left( v_{i}\left( \boldsymbol{\lambda}^{\boldsymbol{'}}\boldsymbol{x}_{\boldsymbol{i}} \right)-v_{j}\left( \boldsymbol{\lambda}^{\boldsymbol{'}}\boldsymbol{x}_{\boldsymbol{j}} \right) \right)\left( \boldsymbol{\lambda}^{\boldsymbol{'}}\boldsymbol{x}_{\boldsymbol{i}}-\theta_{o}x_{i,o} \right)\left( \boldsymbol{\lambda}^{\boldsymbol{'}}\boldsymbol{x}_{\boldsymbol{j}}-\theta_{o}x_{j,o} \right)}{v_{i}\left( \boldsymbol{\lambda}^{\boldsymbol{'}}\boldsymbol{x}_{\boldsymbol{i}} \right)^{2}\left( v_{j}\left( \boldsymbol{\lambda}^{\boldsymbol{'}}\boldsymbol{x}_{\boldsymbol{j}}-\theta_{o}x_{j,o} \right)-v_{i}\left( \boldsymbol{\lambda}^{\boldsymbol{'}}\boldsymbol{x}_{\boldsymbol{i}}-\theta_{o}x_{i,o} \right) \right)} \right].$$

Combining this result with the second half of the formula gives:

$$\sigma_{i,j}^{H}=\left[ \frac{\left( \frac{v_{i}}{v_{j}} \right)}{\left( \frac{\boldsymbol{\lambda}^{\boldsymbol{'}}\boldsymbol{x}_{\boldsymbol{j}}}{\boldsymbol{\lambda}^{\boldsymbol{'}}\boldsymbol{x}_{\boldsymbol{i}}} \right)} \right]\left[ \frac{v_{j}\left( v_{i}\left( \boldsymbol{\lambda}^{\boldsymbol{'}}\boldsymbol{x}_{\boldsymbol{i}} \right)-v_{j}\left( \boldsymbol{\lambda}^{\boldsymbol{'}}\boldsymbol{x}_{\boldsymbol{j}} \right) \right)\left( \boldsymbol{\lambda}^{\boldsymbol{'}}\boldsymbol{x}_{\boldsymbol{i}}-\theta_{o}x_{i,o} \right)\left( \boldsymbol{\lambda}^{\boldsymbol{'}}\boldsymbol{x}_{\boldsymbol{j}}-\theta_{o}x_{j,o} \right)}{v_{i}\left( \boldsymbol{\lambda}^{\boldsymbol{'}}\boldsymbol{x}_{\boldsymbol{i}} \right)^{2}\left( v_{j}\left( \boldsymbol{\lambda}^{\boldsymbol{'}}\boldsymbol{x}_{\boldsymbol{j}}-\theta_{o}x_{j,o} \right)-v_{i}\left( \boldsymbol{\lambda}^{\boldsymbol{'}}\boldsymbol{x}_{\boldsymbol{i}}-\theta_{o}x_{i,o} \right) \right)} \right]$$

$$\sigma_{i,j}^{H}=\left[ \frac{\left( \boldsymbol{\lambda}^{\boldsymbol{'}}\boldsymbol{x}_{\boldsymbol{i}}v_{i}v_{j} \right)\left( v_{i}\left( \boldsymbol{\lambda}^{\boldsymbol{'}}\boldsymbol{x}_{\boldsymbol{i}} \right)-v_{j}\left( \boldsymbol{\lambda}^{\boldsymbol{'}}\boldsymbol{x}_{\boldsymbol{j}} \right) \right)\left( \boldsymbol{\lambda}^{\boldsymbol{'}}\boldsymbol{x}_{\boldsymbol{i}}-\theta_{o}x_{i,o} \right)\left( \boldsymbol{\lambda}^{\boldsymbol{'}}\boldsymbol{x}_{\boldsymbol{j}}-\theta_{o}x_{j,o} \right)}{\left( \boldsymbol{\lambda}^{\boldsymbol{'}}\boldsymbol{x}_{\boldsymbol{j}}v_{j}v_{i}\left( \boldsymbol{\lambda}^{\boldsymbol{'}}\boldsymbol{x}_{\boldsymbol{i}} \right)^{2} \right)\left( v_{j}\left( \boldsymbol{\lambda}^{\boldsymbol{'}}\boldsymbol{x}_{\boldsymbol{j}}-\theta_{o}x_{j,o} \right)-v_{i}\left( \boldsymbol{\lambda}^{\boldsymbol{'}}\boldsymbol{x}_{\boldsymbol{i}}-\theta_{o}x_{i,o} \right) \right)} \right]$$

$$\sigma_{i,j}^{H}=\left[ \frac{\left( v_{i}v_{j} \right)\left( v_{i}\left( \boldsymbol{\lambda}^{\boldsymbol{'}}\boldsymbol{x}_{\boldsymbol{i}} \right)-v_{j}\left( \boldsymbol{\lambda}^{\boldsymbol{'}}\boldsymbol{x}_{\boldsymbol{j}} \right) \right)\left( \boldsymbol{\lambda}^{\boldsymbol{'}}\boldsymbol{x}_{\boldsymbol{i}}-\theta_{o}x_{i,o} \right)\left( \boldsymbol{\lambda}^{\boldsymbol{'}}\boldsymbol{x}_{\boldsymbol{j}}-\theta_{o}x_{j,o} \right)}{\left( v_{i}v_{j}\boldsymbol{\lambda}^{\boldsymbol{'}}\boldsymbol{x}_{\boldsymbol{i}}\boldsymbol{\lambda}^{\boldsymbol{'}}\boldsymbol{x}_{\boldsymbol{j}} \right)\left( v_{j}\left( \boldsymbol{\lambda}^{\boldsymbol{'}}\boldsymbol{x}_{\boldsymbol{j}}-\theta_{o}x_{j,o} \right)-v_{i}\left( \boldsymbol{\lambda}^{\boldsymbol{'}}\boldsymbol{x}_{\boldsymbol{i}}-\theta_{o}x_{i,o} \right) \right)} \right]$$

$$\boldsymbol{\sigma}_{\boldsymbol{i,j}}^{\boldsymbol{H}}=\left[ \frac{\left( v_{i}\left( \boldsymbol{\lambda}^{\boldsymbol{'}}\boldsymbol{x}_{\boldsymbol{i}} \right)-v_{j}\left( \boldsymbol{\lambda}^{\boldsymbol{'}}\boldsymbol{x}_{\boldsymbol{j}} \right) \right)\left( \boldsymbol{\lambda}^{\boldsymbol{'}}\boldsymbol{x}_{\boldsymbol{i}}-\theta_{o}x_{i,o} \right)\left( \boldsymbol{\lambda}^{\boldsymbol{'}}\boldsymbol{x}_{\boldsymbol{j}}-\theta_{o}x_{j,o} \right)}{\left( \boldsymbol{\lambda}^{\boldsymbol{'}}\boldsymbol{x}_{\boldsymbol{i}}\boldsymbol{\lambda}^{\boldsymbol{'}}\boldsymbol{x}_{\boldsymbol{j}} \right)\left( v_{j}\left( \boldsymbol{\lambda}^{\boldsymbol{'}}\boldsymbol{x}_{\boldsymbol{j}}-\theta_{o}x_{j,o} \right)-v_{i}\left( \boldsymbol{\lambda}^{\boldsymbol{'}}\boldsymbol{x}_{\boldsymbol{i}}-\theta_{o}x_{i,o} \right) \right)} \right]$$

**B. Derivation of the elasticity of substitution for inefficient firms (cost problem)**

**B.1. The Hicksian elasticity of substitution**

The cost efficiency problem can be represented as:

*Cost(min):* ${}_{z,\lambda}^{min}{\boldsymbol{w}^{\boldsymbol{'}}}\boldsymbol{z}$

*Subject to:* $z_{k}-\boldsymbol{\lambda}^{\boldsymbol{'}}\boldsymbol{x}_{\boldsymbol{k}}\geq0$ $\forall\text{k inputs}$ $\to\boldsymbol{v}_{\boldsymbol{k}}$

$\boldsymbol{\lambda}^{\boldsymbol{'}}\boldsymbol{y}_{\boldsymbol{m}}-y_{m,o}\geq0$ $\boldsymbol{\forall}\text{m outputs}$ $\to\boldsymbol{u}_{\boldsymbol{m}}$

$\boldsymbol{e}^{\boldsymbol{'}}\boldsymbol{\lambda}=1$

$\boldsymbol{\lambda}\geq0$

Let *L* denote the Lagrangian function for the cost efficiency problem, where $w_{k}$ refers to the *k*^th^ input’s price and $z_{k}$ refers to the *k*^th^ cost-minimizing level of input for the firm.

$$L=w^{'}z+\sum_{k} v_{k}\left( \boldsymbol{\lambda}^{\boldsymbol{'}}\boldsymbol{x}_{\boldsymbol{k}}-z_{k} \right)-\sum_{m} u_{m}\left( \boldsymbol{\lambda}^{\boldsymbol{'}}\boldsymbol{y}_{\boldsymbol{m}}-y_{m,o} \right)+u_{o}\left( \boldsymbol{e}^{\boldsymbol{'}}\boldsymbol{\lambda}-1 \right),$$

The Hicksian elasticity of input substitution of interest for an inefficient firm (i.e. at the cost minimizing level of input use) can be represented as:

$$\sigma_{i,j}^{HC}=\frac{\partial ln\left( \frac{z_{j}}{z_{i}} \right)}{\partial ln\left( \frac{w_{i}}{w_{j}} \right)}=\frac{\partial lnz_{j}-\partial lnz_{i}}{\partial lnw_{i}-\partial lnw_{j}}=\frac{\partial lnz_{j}}{\partial lnw_{i}-\partial lnw_{j}}-\frac{\partial lnz_{i}}{\partial lnw_{i}-\partial lnw_{j}}$$

$$=\left( \frac{\partial lnw_{i}-\partial lnw_{j}}{\partial lnz_{j}} \right)^{-1}-\left( \frac{\partial lnw_{i}-\partial lnw_{j}}{\partial lnz_{i}} \right)^{-1}$$

$$=\left( \frac{\partial lnw_{i}}{\partial lnz_{j}}-\frac{\partial lnw_{j}}{\partial lnz_{j}} \right)^{-1}-\left( \frac{\partial lnw_{i}}{\partial lnz_{i}}-\frac{\partial lnw_{j}}{\partial lnz_{i}} \right)^{-1}$$

$$=\left( \frac{z_{j}\partial w_{i}}{w_{i}\partial z_{j}}-\frac{z_{j}\partial w_{j}}{w_{j}\partial z_{j}} \right)^{-1}-\left( \frac{z_{i}\partial w_{i}}{w_{i}\partial z_{i}}-\frac{z_{i}\partial w_{j}}{w_{j}\partial z_{i}} \right)^{-1}$$

$$=\left( \left( \frac{\partial L}{\partial L} \right)\left( \frac{z_{j}\partial w_{i}}{w_{i}\partial z_{j}}-\frac{z_{j}\partial w_{j}}{w_{j}\partial z_{j}} \right) \right)^{-1}-\left( \left( \frac{\partial L}{\partial L} \right)\left( \frac{z_{i}\partial w_{i}}{w_{i}\partial z_{i}}-\frac{z_{i}\partial w_{j}}{w_{j}\partial z_{i}} \right) \right)^{-1}$$

$$=\left( \frac{z_{j}\left( \frac{\partial L}{\partial z_{j}} \right)}{w_{i}\left( \frac{\partial L}{\partial w_{i}} \right)}-\frac{z_{j}\left( \frac{\partial L}{\partial z_{j}} \right)}{w_{j}\left( \frac{\partial L}{\partial w_{j}} \right)} \right)^{-1}-\left( \frac{z_{i}\left( \frac{\partial L}{\partial z_{i}} \right)}{w_{i}\left( \frac{\partial L}{\partial w_{i}} \right)}-\frac{z_{i}\left( \frac{\partial L}{\partial z_{i}} \right)}{w_{j}\left( \frac{\partial L}{\partial w_{j}} \right)} \right)^{-1}$$

$$=\left( \frac{z_{j}\left( \frac{\partial L}{\partial z_{j}} \right)}{w_{i}z_{i}}-\frac{z_{j}\left( \frac{\partial L}{\partial z_{j}} \right)}{w_{j}z_{j}} \right)^{-1}-\left( \frac{z_{i}\left( \frac{\partial L}{\partial z_{i}} \right)}{w_{i}z_{i}}-\frac{z_{i}\left( \frac{\partial L}{\partial z_{i}} \right)}{w_{j}z_{j}} \right)^{-1},$$

where $\frac{\partial L}{\partial w_{k}}=z_{k} \text{and} \frac{\partial L}{\partial z_{k}}=w_{k}-v_{k}$. Given these relationships:

$$\sigma_{i,j}^{HC}\boldsymbol{=}\left( \frac{z_{j}\left( w_{j}-v_{j} \right)}{w_{i}z_{i}}-\frac{z_{j}\left( w_{j}-v_{j} \right)}{w_{j}z_{j}} \right)^{-1}-\left( \frac{z_{i}\left( w_{i}-v_{i} \right)}{w_{i}z_{i}}-\frac{z_{i}\left( w_{i}-v_{i} \right)}{w_{j}z_{j}} \right)^{-1}$$

**B.2. The Morishima elasticity of substitution**

The cost efficiency problem can be represented as:

*Cost(min):* ${}_{z,\lambda}^{min}{\boldsymbol{w}^{\boldsymbol{'}}}\boldsymbol{z}$

*Subject to:* $z_{k}-\boldsymbol{\lambda}^{\boldsymbol{'}}\boldsymbol{x}_{\boldsymbol{k}}\geq0$ $\forall\text{k inputs}$ $\to\boldsymbol{v}_{\boldsymbol{k}}$

$\boldsymbol{\lambda}^{\boldsymbol{'}}\boldsymbol{y}_{\boldsymbol{m}}-y_{m,o}\geq0$ $\boldsymbol{\forall}\text{m outputs}$ $\to\boldsymbol{u}_{\boldsymbol{m}}$

$\boldsymbol{e}^{\boldsymbol{'}}\boldsymbol{\lambda}=1$

$\boldsymbol{\lambda}\geq0$

Let *L* denote the Lagrangian function for the cost efficiency problem, where $w_{k}$ refers to the k^th^ input’s price, and $z_{k}$ refers to the *k*^th^ cost-minimizing input level for the firm.

$$L=\boldsymbol{w}^{\boldsymbol{'}}\boldsymbol{z}+\sum_{k} v_{k}\left( \boldsymbol{\lambda}^{\boldsymbol{'}}\boldsymbol{x}_{\boldsymbol{k}}-z_{k} \right)-\sum_{m} u_{m}\left( \boldsymbol{\lambda}^{\boldsymbol{'}}\boldsymbol{y}_{\boldsymbol{m}}-y_{m,o} \right)+u_{o}\left( \boldsymbol{e}^{\boldsymbol{'}}\boldsymbol{\lambda}-1 \right),$$

The Morshima elasticity of input substitution at the cost minimizing level of input use can be represented as:

$$\sigma_{i,j}^{MC}=\frac{dln\left( \frac{z_{i}}{z_{j}} \right)}{dln\left( w_{j} \right)}=\frac{dlnz_{i}-dlnz_{j}}{dlnw_{j}}=\frac{dz_{i}}{dw_{j}}\frac{w_{j}}{z_{i}}-\frac{dz_{j}}{dw_{j}}\frac{w_{j}}{z_{j}}=\frac{\left( \frac{dL}{dw_{j}} \right)}{\left( \frac{dL}{dz_{i}} \right)}\frac{w_{j}}{z_{i}}-\frac{\left( \frac{dL}{dw_{j}} \right)}{\left( \frac{dL}{dz_{j}} \right)}\frac{w_{j}}{z_{j}},$$

where $\frac{\partial L}{\partial w_{k}}=z_{k} \text{and} \frac{\partial L}{\partial z_{k}}=w_{k}-v_{k}$. Then:

$$\boldsymbol{\sigma}_{\boldsymbol{i,j}}^{\boldsymbol{MC}}=\frac{w_{j}z_{j}}{\left( w_{i}-v_{i} \right)z_{i}}-\frac{w_{j}z_{j}}{\left( w_{j}-v_{j} \right)z_{j}}$$
